# User preference-based human-in-the-loop tuning of exoskeleton assistance during walking

**DOI:** 10.1038/s44385-026-00085-7

**Published:** 2026-05-13

**Authors:** Niklas Schäfer, Guoping Zhao, Bowen Li, Mario Kupnik, André Seyfarth, Philipp Beckerle, Martin Grimmer

**Affiliations:** 1 Measurement and Sensor Technology Group, Technische Universität Darmstadt, Darmstadt, Germany; 2 School of Mechanical Engineering, Southeast University, Nanjing, China; 3 Department of Fluid Control and Automation, Harbin Institute of Technology, Harbin, China; 4 Lauflabor Locomotion Laboratory, Technische Universität Darmstadt, Darmstadt, Germany; 5 Chair of Autonomous Systems and Mechatronics, Friedrich-Alexander-Universität Erlangen-Nürnberg, Erlangen, Germany; 6 Control and Cyber-Physical Systems Laboratory, Technische Universität Darmstadt, Darmstadt, Germany

**Keywords:** Engineering, Health care, Neuroscience

## Abstract

Personalized exoskeleton assistance has substantial potential to enhance human locomotion performance. However, current human-in-the-loop optimization methods for generating personalized assistance are cumbersome and time-consuming. Since humans can perceive locomotion through internal sensory feedback, user preference-based self-tuning may facilitate the individualization of exoskeleton assistance to meet individual needs. Here, we explore a user-driven human-in-the-loop tuning approach for walking assistance, hypothesizing that individuals can quickly find their preferred personalized assistance through subjective perception. We conducted experiments with 11 healthy participants, who were instructed to tune four control parameters while wearing a hip exoskeleton and walking on a treadmill. The tuning procedure concluded when participants indicated that they had found their preferred assistance. Then we surveyed the sense of agency to assess the user experience. We evaluated the effort of walking with the preferred setting and explored the metabolic cost landscape around it. Participants identified their preference in 10.9 ± 0.9 min, while testing 30.5 settings and spending 18.7 s per setting on average. Preferred assistance profiles varied widely between participants, with timing differences of up to 22.5% of the stride time. The metabolic cost of walking with the preferred assistance was reduced by 16.6 ± 1.1% compared to walking with the exoskeleton in a zero-torque condition. Timing deviations of up to ±8% of the stride time did not significantly affect metabolic cost reduction, indicating the robustness of the preferred assistance profiles. Significant changes in the sense of agency between unassisted and assisted walking demonstrate its sensitivity to partial exoskeleton assistance. The results highlight the potential of preference-based user-tuning while suggesting that additional guidance throughout the user-tuning procedure may support a systematic exploration, thereby advancing the preference-based individualization of exoskeleton assistance.

## Introduction

Lower limb exoskeletons hold great potential to improve rehabilitation as well as to assist users in daily live^1–3^. They have been shown to provide assistance to users with and without gait impairments, for instance by increasing gait symmetry^4^ and self-selected walking speed^5^, supporting physical exercise^6–8^, as well as reducing the metabolic cost during walking^9–12^, running^13,14^, and cycling^15^. However, the efficacy of many exoskeletons remains limited^2^. A major challenge is the interdependent nature of the human-machine interaction, which requires appropriate control. Conventional approaches for determining effective control parameters include the replication of biomechanical characteristics such as joint torque^11,16^, often based on data taken from literature, as well as biomechanical simulation and modeling^17,18^. However, due to individual differences among users, individualization is necessary to achieve optimal assistance^10,19–21^.

Human-in-the-loop experiments are a promising human-centered approach to systematically investigate the interactions between humans and exoskeletons^22^. Human-in-the-loop optimization (HILO), in particular, is able to generate individualized assistance^9,10,21^. HILO iteratively optimizes a set of control parameters in order to minimize or maximize an objective function based on human-centered metrics^23,24^. Most often, the objective function is based on the metabolic cost in order to minimize the user’s effort^25^, and several studies have demonstrated the potential of HILO to achieve significant reductions^9,10,26^. However, the relatively long optimization procedure restricts the approach’s practicability, especially for the elderly and individuals with gait impairments. The slow physiological dynamics and the measurement noise are the main limitations of metabolic cost acquisition, which current research attempts to overcome by metrics based on alternative feedback variables, such as electromyography^27,28^, joint kinematics^29^, or a combination of multiple physiological signals^30^. Another potential drawback is the reliance on a single measure, which could potentially be improved by including multiple metrics in the objective function^31–33^. However, even if all relevant factors were known and measurable, it would still be an open question how to weight them in the objective function. Another challenge is that, from a user’s perspective, the optimal or preferred assistance might depend not only on technical and biomechanical aspects but also on experiential ones, such as comfort or perception of safety^34^. Therefore, the development of assistance systems is a multifaceted challenge that includes a functional performance perspective as well as a user experience perspective^35^.

User experience is defined as a person’s perceptions and reactions resulting from the usage of a system and depends on the user’s internal state, the characteristics of the system, as well as the interaction context^36^. The sense of agency (SoA) is a subcomponent of embodiment^37^ and an important aspect of user experience in the context of human-machine interaction^38^, especially when the technology has a direct influence on the body^39^. The SoA refers to the experience of being in control of one’s own actions, which is assumed to arise based on the intention to execute an action, the expectation of the corresponding sensory outcome, and the experienced sensory outcome^40^. Thus, the SoA is thought to contribute to the separation of own movements and those induced by external influences. So far, SoA research focuses on goal-directed movements of the upper limbs^38,39,41,42^, but there have also been studies investigating SoA in the context of walking^43–45^. In a study investigating physical human-machine interaction, participants reported a stronger SoA when being properly assisted, which emphasizes the assumption that concepts from psychology can contribute to a human-centered validation of control designs^42^. However, SoA and user experience in general are challenging to measure or even to assess in a way that is applicable to HILO.

The human body itself contains various sensory organs whose outputs are fused and contribute to a certain user experience. Moreover, while there are trade-offs with other objectives, such as saving time^46^ or avoiding local muscle fatigue^47^, humans generally prefer to move in ways that minimize the metabolic cost^48,49^, and they are able to continuously adapt to new conditions^49^. In line with these trade-offs, previous research has shown that user-preferred exoskeleton assistance does not necessarily correlate with metabolic cost reduction^19^. Therefore, user preference might serve as an objective function for control optimization^50^, taking into account the multifaceted nature of the human-exoskeleton interaction. Furthermore, user preference-based individualization does not depend on cumbersome measurement setups, such as respirometry or marker-based motion capture, which is expected to facilitate the practicality of the approach.

The feasibility of humans self-tuning control parameters to find their individual optimum has been demonstrated in a study where participants self-tuned the magnitude and the timing of peak torque while walking with an ankle exoskeleton^51^. Although being blinded to the control parameters, the participants reliably identified their preferred assistance profile relying solely on their perception, underscoring the repeatability of human preference identification. While previous studies have highlighted the potential of user preference-based individualization of exoskeleton assistance^50,51^, they did not evaluate the effect on metabolic cost, which remains an important performance metric. Furthermore, to advance the understanding of user preference-based assistance tuning, it is essential to confirm the approach’s applicability for assistance at other joints and to analyze the impact of the user-tuning procedure and interface.

**Fig. 1:**
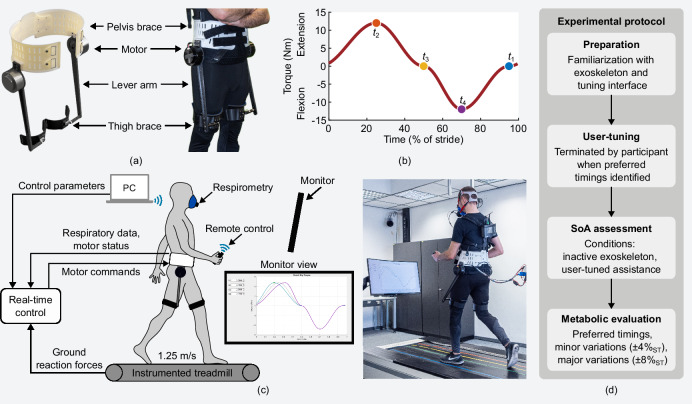
Hip exoskeleton, experimental setup, and protocol. **a** The two electric motors of the bilateral hip exoskeleton generate extension and flexion torques in parallel to both hip joints. **b** The exoskeleton torque profile consists of four half-cycle sinusoidal curves connected at their respective peaks. Control parameters *t*_*i*_ are the timings of the peaks and the zero-crossings between extension and flexion. **c** Participants self-tuned the exoskeleton assistance by adjusting the stride time (ST) of the parameters with a remote control while walking on an instrumented treadmill. **d** User-tuning was terminated when the participants felt that they had found their preferred setting. The sense of agency (SoA) was assessed using a questionnaire. Metabolic evaluation consisted of four sessions testing the preferred setting and variations of these timings.

In this study, we investigate a user-tuning method for individualizing the torque profile of a hip exoskeleton (Fig. 1(a, b)). We develop a tuning interface based on a thumbstick remote control to tune four control parameters, defining the timing of the transitions between extension and flexion torque and the timing of the peak torques^52^. We conduct an experiment with 11 participants without gait impairments using the developed preference-based user-tuning method while walking with the hip exoskeleton (Fig. 1(c, d)). We expect that the participants are able to self-tune the exoskeleton’s hip torque profile to a preferred setting in a time-efficient manner. In addition, we evaluate the effort of walking with the preferred setting and explore the metabolic cost landscape around it. Furthermore, in order to advance the understanding of the user-tuning approach, we investigate methodological characteristics of the user-tuning procedure, namely temporal aspects (total tuning duration, time per setting), exploration step sizes between successive settings, and solution space coverage (number of settings, range of settings). Finally, we assume that the SoA can be used to assess the user experience in the context of human-exoskeleton interaction. We investigate whether the SoA is sensitive to hip exoskeleton assistance, expecting only a slight decrease while walking with user-tuned assistance compared to walking without assistance. The results of this study can inform the design of future user-tuning approaches and demonstrate the potential of preference-based user-tuning for a time-efficient individualization of lower limb exoskeleton assistance.

## Results

### Preferred setting

**Fig. 2:**
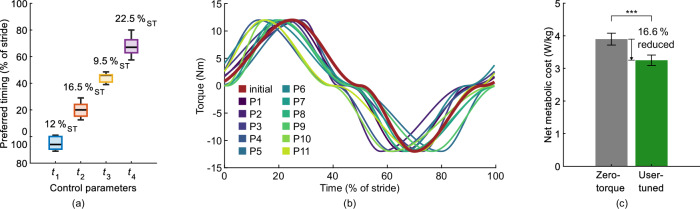
User-tuning results. **a** Ranges of the preferred control parameter timings. Numerical values above box plots indicate the total ranges. **b** User-tuned hip torque profiles. The preferred assistance varies widely between participants (P1 to P11). **c** Net metabolic rates of walking with the inactive exoskeleton (zero-torque) and the user-tuned assistance torque. Bars are means, error bars are standard errors, and triple asterisks denote statistical significance with *p* < 0.001 (Wilcoxon signed-rank test).

The four control parameters *t*_1_ to *t*_4_, denoting timings in the gait cycle, are expressed as percentages of the stride time (%_ST_, see “Methods”). The average preferred timings for *t*_1_ to *t*_4_ are 95.3%_ST_, 20.0%_ST_, 44.2%_ST_, and 68.1%_ST_ with respect to the heel strike, respectively. The user-tuned torque profiles vary widely across participants (Fig. 2(a, b)). The preferred timings for *t*_3_ show the smallest variability with an interquartile range (IQR) of 5.5%_ST_, which is 38% to 44% smaller relative to the IQRs of the other timings. In addition, *t*_3_ shows the smallest total range.

The mean net metabolic cost of walking with the user-tuned assistance (3.3 ± 0.2 W kg^−1^, mean ± standard error) is significantly lower than walking with the exoskeleton in zero-torque mode (3.9 ± 0.2 W kg^−1^; Wilcoxon signed-rank test, *W* = 0, *p* < 0.001; Fig. 2(c)). The average net metabolic reduction is 16.6 ± 1.1%, spanning from 12.2 ± 2.3% to 21.6 ± 2.3% for individual participants.

**Fig. 3:**
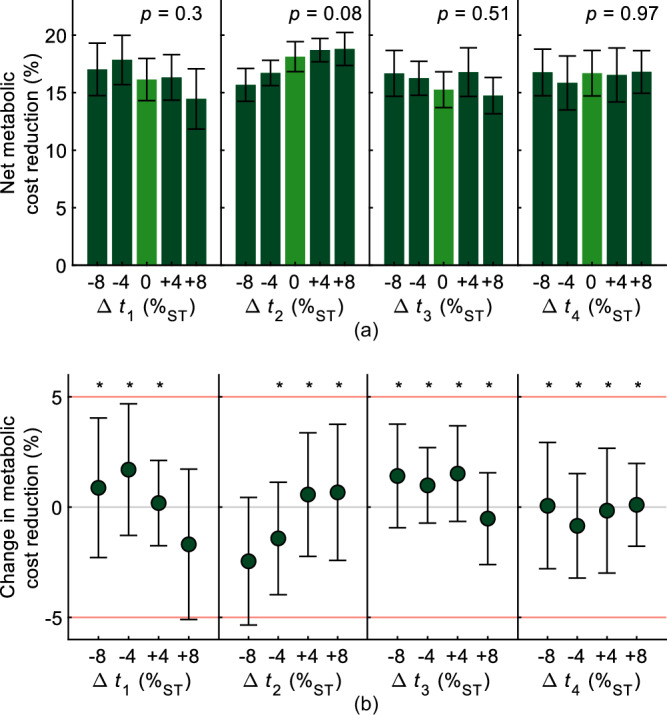
Metabolic evaluation of the regions around the preferred assistance. Timing variations Δ*t*_*i*_ of ±4%_ST_ and ±8%_ST_, respectively, were added to the preferred timings (Δ*t*_*i*_ = 0%_ST_). **a** Metabolic cost reduction. Repeated measures ANOVA does not indicate statistically significant differences (*p* > 0.05 for all control parameters). Bars are means, and error bars are standard errors. **b** Equivalence testing of change in metabolic reduction due to assistance timing variations. In all but two conditions, the change of metabolic cost lies in the equivalence zone ±5%. Error bars indicate the 90% confidence interval. Asterisks denote statistical equivalence, i.e., *p* < 0.05 for both one-sided *t*-tests.

The tested timing variations of ±4%_ST_ and ±8%_ST_ do not lead to a significant effect in the metabolic cost reduction for any of the four control parameters (repeated measures analysis of variance (ANOVA), *p*_*t*1_ = 0.3, *p*_*t*2_ = 0.08, *p*_*t*3_ = 0.51, *p*_*t*4_ = 0.97; Fig. 3(a)). The mean net metabolic cost reduction in all but two major variations can be considered statistically equivalent to the reduction achieved with the preferred timings, using equivalence bounds of ±5% (Fig. 3(b)).

### User-tuning procedure

**Fig. 4:**
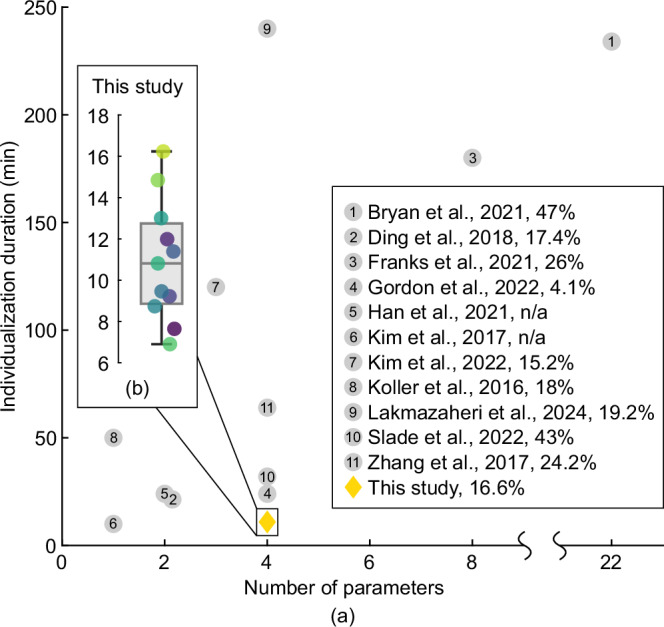
Tuning duration. **a** Comparison with human-in-the-loop optimization approaches from the state of the art regarding individualization duration and number of control parameters. Percentages denote the average metabolic reduction achieved if available. **b** Durations required for self-tuning the preferred hip torque assistance profile in this study. Colored markers denote the individual participants.

All participants found their preferred assistance torque profile within a maximum of 16.2 min ($10.9\pm 0.9\,\min$; Fig. 4(b)). In order to put this result into context, it can be examined in relation to studies that individualized control parameters using HILO, considering both the duration of the customization procedure and the number of control parameters (Fig. 4(a)).

**Fig. 5:**
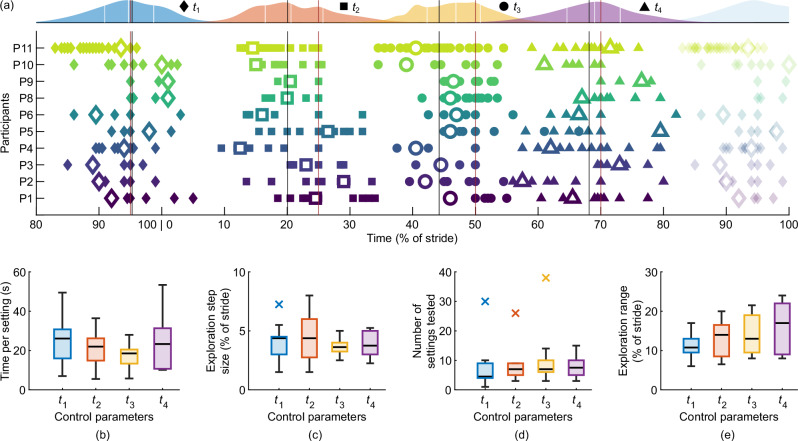
User-tuning procedure. **a** Timings explored by the participants. Colored symbols depict the settings tested, with the preferred setting highlighted by larger unfilled symbols. The colored curves above show the average distribution, with white vertical lines indicating the quartiles. The other vertical lines represent the initial setting (red) and the average of the preferred settings (black) for each parameter. Characteristics are **b** the average time participants spent testing a setting, **c** the average exploration step size, **d** the number of settings tested, and **e** the ranges of settings tested. Participant averages were calculated using the median. The data collected during the tuning procedure of P7 was lost due to a technical issue.

The average time participants spent on testing a setting is 18.7 ± 6.2 s (mean ± standard deviation), which corresponds to 39.1 ± 12.4 steps. Overall, the time per setting spans from 2 s to 126 s. The average time per setting tends to be shorter for *t*_3_ (Fig. 5(b)).

The exploration step sizes aggregated across all participants span from 0.5%_ST_ to 21.5%_ST_. On average, the individual participant minima and maxima are 0.65%_ST_ and 13.25%_ST_, respectively. The medians of the average step sizes are between 3.6%_ST_ and 4.4%_ST_, and thus similar for all parameters, but the IQR is smaller for *t*_3_ (Fig. 5(c)).

The number of settings tested during the user-tuning procedure spans from 16 to 111 (median = 30.5, IQR = 11). The number of settings tested tends to be lower for *t*_1_ (median = 4.5), while it is similar for the other three timings (median ∈ [7, 7.5]; Fig. 5(d)). Although the tuning interface allowed adjusting multiple timings before applying the new assistance profile to the exoskeleton, the participants changed only one parameter at a time in 97.5% of all cases.

The average ranges of the timings explored by individual participants are slightly increasing from *t*_1_ to *t*_4_ with 11.2%_ST_, 13.3%_ST_, 14%_ST_, and 16%_ST_ (Fig. 5(e)). The total exploration ranges aggregated across all participants are about double the average individual ranges with 22%_ST_, 24.5%_ST_, 32%_ST_ and 26%_ST_ for *t*_1_ to *t*_4_. While for the other parameters the average preference is in the center of the solution space explored, for *t*_3_ it is slightly asymmetric with a 2.7%_ST_ shift to an earlier timing (Fig. 5(a)).

### Sense of agency

**Fig. 6:**
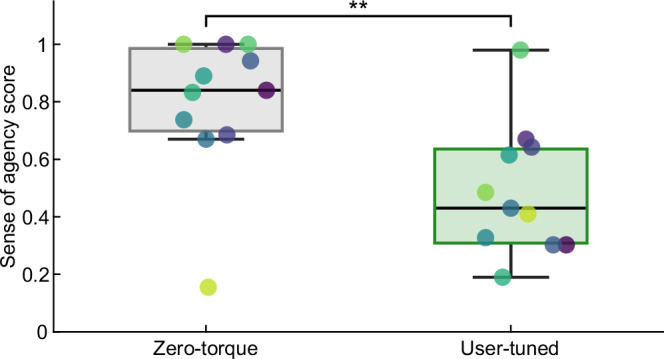
Sense of agency assessment. For all but one participant, the sense of agency score was reduced, indicating a decrease in perceived control, when assisted with the user-tuned torque profile compared to the zero-torque condition. Colored markers denote the individual participants. Double asterisks denote statistical significance with *p* < 0.01 (paired samples *t*-test).

For all but one participant, the zero-torque condition results in a SoA score ≥0.67 out of 1 (0.80 ± 0.07). In the assisted condition, the SoA score is reduced (0.49 ± 0.07; paired samples *t*-test, *p* = 0.002; Fig. 6). One participant exhibited the opposite behavior, with the SoA score in the zero-torque condition being lower than in the assisted condition.

## Discussion

### Preferred setting

Similar to previous HILO studies^9,10,21,53^, we found that the preferred torque profiles varied widely across participants (Fig. 2(a, b)). This supports the assumption that exoskeleton users prefer an individualized assistance profile^51^. However, the smaller variation in the user-tuned *t*_3_ compared to the other timing parameters suggests that a narrow parameter range, or even a fixed value, could be used for *t*_3_ to further reduce the tuning duration in future user-tuning approaches.

We did not expect the participants to optimize the exoskeleton assistance in terms of walking effort, still, metabolic cost evaluation can be considered the gold standard for the assessment of assistance efficacy^2^. In particular, HILO studies minimizing the metabolic cost of walking may serve as an indication of what is achievable in this regard. While the objectives differ and the approaches are not directly comparable, this perspective helps to put our results into context. The average reduction in metabolic cost achieved by our user-tuning approach was 16.6% compared to walking with the inactive exoskeleton. We estimate the additional metabolic cost caused by the exoskeleton’s mass (2.5 kg at the waist, 0.5 kg at the thighs) to be 0.15 W kg^−1^ ^54^. This translates to a 13.1% reduction in metabolic cost compared to walking without the exoskeleton, which is within the medium range of previous hip exoskeleton studies^10,12,19,31,53,55^. Differences may be due to manifold reasons, including the individualization approach, the assistance magnitude, as well as the participants’ experience in using the exoskeleton.

It has been shown that an increased assistance magnitude can result in larger metabolic reductions^16^. In our study, the peak torque was set to 12 N m, in line with other hip exoskeleton studies^19,53^, to balance effective assistance with user comfort. This value corresponds to about 17% of the peak hip extension torque during human walking^56^, thus a larger assistance magnitude is conceivable. However, at some point, this may compromise comfort, and consequently user preference, and previous research has demonstrated that simply increasing the assistance magnitude does not necessarily lead to larger metabolic reductions^57^. In addition, previous research suggests that assistance with extension and flexion peak torques of up to 12 N m could potentially already achieve a metabolic reduction of 20% to 25% compared to not wearing the hip exoskeleton^55^.

Previous exoskeleton studies have demonstrated that larger metabolic reductions can be achieved with additional training. For example, two 30 min training sessions over the course of the previous 7 to 14 days resulted in an additional reduction of 8.6%^58^. However, full adaptation may take some time, as demonstrated in a study where participants required about 109 min of walking over the course of multiple days before further training yielded no significant additional reduction^20^. All but one of the aforementioned hip exoskeleton studies reported that their participants had experience walking with the exoskeleton prior to the day of the experiment^10,19,31,55^. Although our participants gathered some experience over the course of the experimental protocol (up to 111 min), they had no prior experience with exoskeleton-assisted walking. Therefore, we expect that with additional training, larger reductions in metabolic cost can be achieved. Altogether, the preference-based user-tuning based on the given initial timings seems reasonable to accomplish customized assistance that results in metabolic reductions similar to those achieved with sensor-based individualization approaches such as HILO.

The deviation between the observed metabolic cost reduction and the potentially achievable reduction indicates the participants did not tune the assistance based on metabolic cost alone, which aligns with previous observations that user-preferred exoskeleton assistance does not necessarily correlate with metabolic cost reduction^19^. This is further underlined by our exploration of the metabolic cost landscapes around the preferred settings. Previous studies have suggested that a metabolic optimum can be identified by increasing metabolic cost when varying the assistance timing, forming a U-shaped pattern^19,55^, which is not observed in this study (Fig. 3(a)). We assume that the participants incorporated additional metrics, such as comfort, into their internal objective function. The observation that the reduction in all but two conditions can be considered statistically equivalent (Fig. 3(b)) might indicate that users prefer a certain degree of robustness, i.e., the benefit remains rather constant despite deviations in the assistance profile. However, there may be further reasons why no significant change is observed. Analyzing previous studies suggests that there is not only a dependency on the extent of the variation, but also on the number of parameters changed simultaneously^19,55,59^, thus larger variations or varying multiple timings at once might have been necessary to observe significant changes in metabolic cost. Moreover, the metabolic cost landscapes of the participants might have been relatively flat in the tested ranges, resulting in metabolic cost changes below the just noticeable difference^60^, and a larger number of participants might have yielded clearer results, as measuring metabolic cost using indirect calorimetry is inherently noisy.

In order to advance the understanding of how the user preference relates to walking effort minimization, we compare our results to hip exoskeleton studies that employed HILO with a metabolic cost-based objective function^10,21,31^. Ding et al.^10^ optimized extension assistance, and the resulting timings corresponding to *t*_2_ and *t*_3_ were 21.1%_ST_ and 38.7%_ST_, respectively. Complementary, Kim et al.^21^ optimized flexion assistance and the resulting timings corresponding to *t*_1_ and *t*_4_ were 96%_ST_ and 75.3%_ST_, respectively. Compared to these two studies, our average timings for *t*_1_ to *t*_4_ show differences of −0.7%_ST_, −1.1%_ST_, 5.5%_ST_, and −7.2%_ST_, respectively, with negative values indicating that our timing occurred earlier in the gait cycle. In Franks et al.^31^ extension and flexion assistance were optimized simultaneously, leading to average optimized timings of 92.2%_ST_, 10.2%_ST_, 32.6%_ST_, and 65.9%_ST_ corresponding to *t*_1_ to *t*_4_, respectively. The differences to our results are 3.1%_ST_, 9.8%_ST_, 11.6%_ST_, and 2.2%_ST_. Based on these comparisons, we conclude that our preference-based results are within reasonable ranges to reduce the metabolic cost of walking. Only for *t*_3_, Kim et al.^21^ and Franks et al.^31^ consistently identified earlier timings. For *t*_3,_ our initial setting was quite late within the gait cycle, considering the optimized timings of the other studies, however, participants clearly preferred settings that were closer to these earlier timings. Altogether, the average preferred timings tending towards HILO-based optima highlight the potential of user preference-based tuning to reduce walking effort while simultaneously accounting for other factors important to the user.

### User-tuning procedure

We investigated methodological characteristics of the user-tuning procedure including temporal aspects (total tuning duration, time per setting), the exploration step sizes between successive settings, and the solution space coverage (number of settings, range of settings).

Despite involving four control parameters, our user-tuning approach required only a fourth of the time compared to using HILO to individualize two parameters in Ding et al.^10^ and half the time compared to using HILO to individualize four parameters in Gordon et al.^53^. However, neither individualization protocol included a real-time convergence criterion. Still, when the convergence time is extracted post hoc, our average total tuning duration is half of the convergence time in Ding et al.^10^ and slightly shorter than in Gordon et al.^53^. In contrast to our approach using subjective user feedback, Ding et al.^10^ used respiratory measurements and Gordon et al.^53^ used kinematic and external force measurements to estimate the metabolic cost. Nevertheless, when comparing to other studies employing HILO for individualizing exoskeleton assistance (Fig. 4(a)), the user preference-based tuning emerges as a time-efficient solution, especially when considering the number of control parameters. This time efficiency is expected to facilitate practical applicability, e.g., by enabling quick retuning in response to changes in the user preference. Moreover, a time-efficient solution might be particularly beneficial for certain target populations, such as the elderly or individuals with gait impairments. Therefore, combining the time efficiency of user-tuning with HILO appears promising. For example, user-tuning could narrow down the solution space by quickly identifying the preferred region, followed by a brief HILO protocol for fine-tuning with regard to a specific objective.

The average time per setting in our study was about three to four times as long as in Ingraham et al.^51^, where participants self-tuned ankle exoskeleton assistance by manually selecting different settings. It was also about two-thirds compared to Lee et al.^50^, where participants recurrently compared two recommended assistance profiles, assuming equally shared times for the conditions compared. Differences in time per setting may be due to different designs of the user-tuning approaches. It is reasonable to assume that a manual exploration approach requires more time per setting than an exploration based on recommendations due to the additional time required for manually selecting settings. However, intuitive user interface design can expedite manual adjustments, as demonstrated by the fast iterations in Ingraham et al.^51^. In addition, the time per setting may scale with the number of control parameters, as there are more options to choose from and more variations between assistance profiles to compare. Thus, tuning two parameters in Ingraham et al.^51^ could have been inherently less time-consuming compared to our study involving four parameters. Compared to Lee et al.^50^, the time per setting in our study was shorter, despite involving manual exploration while tuning the same number of control parameters. Considering that the participants in our study mostly adjusted only one parameter at a time, the shorter time per setting can be attributed to less variation between the successive assistance profiles. We did not separately measure times for making adjustments and testing the adjusted setting, but we recommend focusing on such an analysis in future studies to reduce manual adjustment times. Finally, the instructions given to the participants may have further influenced the time per setting. Ingraham et al.^51^ instructed participants to take at least three steps before changing the setting, whereas we instructed participants to perform at least five to ten steps, and Lee et al.^50^ encouraged participants to test both settings as often as necessary to choose their preference. Based on Ingraham et al.^51^ and our study, times of about 5 s to 20 s per setting appear to be necessary for manual exploration, which provides a reasonable point of reference regarding time requirements for the design of future studies.

The exploration step sizes were similar across all four timings, with averages of 4%_ST_. Initially, we expected a decreasing exploration step size over the tuning progress as a sign of convergence, however, this was not observed. Potentially, the interdependence of the timing parameters requires continuous readjustment including various step sizes, thereby preventing the observation of the expected decrease. Still, eight participants made use of the lower resolution limit of 0.5%_ST_, which could have been used to fine-tune the assistance. However, such small timing differences are hard to perceive^61^. The largest step size observed was 21.5%_ST_. We question the usefulness of such small and large adjustments and assume that participants aimed to get experience with different step sizes. Furthermore, we would have expected larger averages and IQRs for *t*_2_ and *t*_3_ due to considerable differences between the initial condition and the average user-tuned result for these timings. While for *t*_2_ results on the IQR were as expected, it was the opposite for *t*_3_ where by far the smallest IQR was observed (Fig. 5(c)). Notably, *t*_2_ represents the peak extension torque while *t*_3_ represents the transition from extension to flexion torque. It is conceivable that *t*_3_ is easier to perceive, as elaborated later, resulting in less variation during exploration.

On average, participants tested 30.5 settings during the tuning procedure. This is slightly higher than the 25.6 settings tested in the first trial block of Ingraham et al.^51^. While we anticipated an increased number of settings tested due to the double number of parameters to tune, the observed increase appears moderate. Examining the individual number of settings, it can be observed that one participant tested 111 settings, while all other participants tested ≤ 36. However, this participant iterated with a reduced average time per setting of 6.4 s, resulting in a comparable total tuning duration as found for all other participants. This observation demonstrates that individual users can approach such tuning tasks differently. When examining the number of settings tested for the four individual timings, the median for *t*_1_ is about two-thirds compared to the other three timings. Given the proximity of the initial setting to the average user preference, it seems plausible that exploring this parameter required fewer settings.

The average range of explored timings did not differ significantly between control parameters. Interestingly, the total exploration range aggregated across all participants was about double the average range explored individually. This is in line with the observation that some participants only partially explored the solution space for certain parameters, including instances where exploration was unilateral regarding the initial timing. Our user-tuning approach assumes that the users have some intuition to distinguish between beneficial and detrimental timings, thus, we did not expect an evenly distributed coverage of the total possible range for each participant. Nevertheless, unilateral exploration limits the range of experienced timings considerably. Furthermore, in cases where participants identified a preferred setting near their exploration limit, it may have been useful to explore even further in this direction.

After analyzing the methodological characteristics of the user-tuning procedure, we found that several aspects stand out for the timing *t*_3_. We found that *t*_3_ has less variability in the preferred timings (Fig. 2(a)), a smaller median and IQR for the average time per setting (Fig. 5(b)), and the smallest IQR for the average exploration step size (Fig. 5(c)). In addition, neither the range nor the number of settings tested exceeded those of the other timings (Fig. 5(d, e)), despite *t*_3_ having the largest difference between the initial setting and the average preferred setting (Fig. 5(a)). It appears that participants have a greater intuition for tuning *t*_3_, potentially due to an increased sensitivity. We suppose that perceiving a change in the exoskeleton’s torque direction is easier than identifying a peak value, as an inaccurate transition timing might oppose the natural movement of the hip. These changes in torque direction are defined by the zero-crossing timings *t*_1_ and *t*_3_ occurring at the end of the swing and during stance, respectively. When the foot is in contact with the ground during the stance phase, potentially a higher number of mechanoreceptors contribute to the perception of the human-exoskeleton interaction forces and torques. Therefore, we assume that the sensitivity of perceiving the exoskeleton assistance, and thereby the timing *t*_3_, is increased during the stance phase.

The analysis of the methodological characteristics of the user-tuning procedure emphasizes certain important aspects. In several cases, participants only partially explored the solution space. In addition, participants predominantly adjusted only one setting at a time, and no convergence in the exploration step size was observed. Furthermore, one participant tested a considerably higher number of settings but spent only a short amount of time per setting, whereas other participants explored only a limited number of settings for certain parameters. Based on these findings, we expect that additional guidance during the tuning procedure can ensure a more systematic exploration. This guidance may include adjusting multiple control parameters simultaneously, adapting exploration step sizes based on the tuning progress, and implementing criteria such as minimum requirements for the number of settings and time per setting. We can imagine different levels of guidance, ranging from the system providing information or recommendations during the user’s manual exploration to automated exploration based on user feedback. Depending on the optimization target, the feedback could be collected through brief questionnaires or by selecting the preferred setting among several recommendations. The latter was demonstrated in a semi-automated HILO approach where users iteratively compared two assistance settings recommended by an optimization algorithm^50^. Initial values for such guided approaches could rely on our findings on the average preference, while for future studies investigating unguided user-tuning, we would recommend randomizing the initial control parameter settings in order to avoid an exploration bias. Appropriate guidance during user-tuning is anticipated to enhance the overall efficiency of the procedure, thereby maximizing the quality of the outcome within a given time frame.

### Sense of agency

As expected, the SoA was lower when using the exoskeleton in the user-tuned condition compared to the zero-torque condition, which shows that SoA is sensitive to the assistance. The SoA was significantly reduced from 0.8 in the zero-torque condition to 0.49 in the user-tuned condition using torque profiles with peaks of 12 N m, which is about 17% of the peak hip extension torque during human walking^56^. This demonstrates that even partial assistance can result in a significant reduction of perceived control. SoA scores of about 0.8 are reasonable for a condition with full human control^42^ and can be classified as unimpaired, thus, the SoA scores observed in the zero-torque condition indicate that the inactive exoskeleton does not excessively restrict the user during walking. In a machine-assisted reaching task, the SoA was reduced by 0.08 and 0.2 for correct and incorrect assistance, respectively, compared to no assistance^42^. Comparing these differences to the 0.31 difference between the zero-torque and user-tuned condition, it appears that there is potential for enhancing the hip exoskeleton assistance in favor of a higher SoA. For a seamless interaction in terms of SoA, it is assumed that the user needs to form an appropriate internal model representing the coupled dynamics of their own body and the exoskeleton^62^. It appears plausible that the limited amount of time spent interacting with the exoskeleton was insufficient to achieve this, given that the participants had no prior experience with the exoskeleton and that the SoA assessment was conducted rather early in the experimental protocol. However, the SoA might also be positively impacted by providing additional information^63^ or a more intuitive controller to facilitate faster adaptation^62^. Altogether, the SoA being sensitive to the hip exoskeleton’s assistance indicates that it might be used as a user experience-related measure in the context of lower limb exoskeleton assistance.

Our study was not designed to evaluate a correlation between SoA and assistance quality. For such an evaluation, studies assessing the SoA under several different torque profiles are required. In particular, it would be important to identify factors, such as balance and interaction forces, that impact the SoA and to evaluate their respective contributions, facilitating cognitive modeling of human body experience^64^. In addition, studies including participants with different levels of exoskeleton experience or assessing the SoA at different stages of exoskeleton training might contribute to a deeper understanding of the underlying adaptations. Such knowledge would enable a more detailed assessment and enhancement of assistance quality, considering not only biomechanical measures but also the SoA as an integral aspect of the user experience perspective. In particular, the SoA questionnaire, comprising only four items, may be a time-efficient solution that allows for iterative feedback in human-in-the-loop experiments.

### Methodological considerations

We did not evaluate the metabolic cost of walking without the exoskeleton. Instead, the reduction in metabolic cost compared to walking without the exoskeleton was estimated by considering the additional metabolic effort due to the exoskeleton’s mass, as has been done in previous studies^21^, confirming the significance of the reductions achieved. However, it has to be noted that user preference and the observed outcomes may differ depending on the physical and cognitive state of the user. We decided not to randomize the initial setting in order to increase the comparability of the individual tuning procedures. While this might have introduced an exploration bias, the high variability in the preference outcomes suggests a rather negligible impact. Furthermore, observations regarding the individual tuning procedures, such as partial exploration or participants predominantly adjusting only one parameter at a time, remain valid despite the possibility of exploration bias. In addition, due to time restrictions, our metabolic evaluation tested only a subset of possible variations around the preferred solutions by varying one timing at a time. Changing multiple timings at once is expected to show increased effects on the metabolic cost of walking.

The main challenge in equivalence testing is to determine the equivalence bounds^65^. There is no established consensus in the literature regarding equivalence bounds for metabolic cost, but some authors have suggested ±10% ^66–68^. In our analysis, we decided to set the equivalence bounds to ±5% for a more restrictive testing, while taking into account findings where average reductions of about 5% were not statistically significant^19,53^. However, since the equivalence bounds are subject to some degree of subjectivity, a slight violation does not necessarily invalidate the assumption of equivalence.

### Conclusions

In this human-in-the-loop study, we proposed a preference-based user-tuning approach to rapidly personalize walking assistance provided by a hip exoskeleton. The results show that all participants were able to find their preferred individualized assistance settings. Although participants did not solely tune the assistance to minimize walking effort, the preferred assistance profiles achieved a comparable level of metabolic cost reduction as found in other hip exoskeleton studies, demonstrating the potential of preference-based assistance individualization to reduce metabolic cost alongside accounting for other user-centered objectives. Moreover, finding the preferred assistance using our user-tuning approach required substantially less time compared to state-of-the-art HILO approaches aimed at minimizing metabolic cost. The metabolic evaluation of the preferred timings, including variations up to ±8%_ST_, did not lead to significant changes in the metabolic cost reduction, indicating robustness of the preferred solutions against timing deviations. The examination of the user-tuning procedure suggests that incorporating additional guidance during exploration of the solution space has the potential to improve both the effectiveness and efficiency of the proposed approach. Furthermore, the analysis of the SoA reveals its sensitivity to hip exoskeleton assistance, suggesting its potential as a valuable user experience-related metric in lower limb exoskeleton design and control.

Based on these findings, we recommend a semi-automated HILO approach that includes user feedback, potentially combining it with sensor-based objectives. By algorithmically controlling the exploration of the solution space, we anticipate advantages due to a more systematic procedure, especially when tuning multiple interdependent control parameters. Moreover, we see questionnaires, such as the SoA assessment, as a complementary tool to biomechanical evaluation for the investigation of factors that determine the user preference. Future work may incorporate additional subjective measures, e.g., comfort or perceived exertion, to further characterize user experience. Altogether, the results of this study have substantial implications for advancing user-tuning methods, enabling holistic customization of exoskeleton assistance to individual needs.

## Methods

### Exoskeleton hardware and control

The study was conducted using a bilateral hip exoskeleton (Fig. 1(a)). The exoskeleton interfaces with the user by a pelvis brace and by a brace above the knee at each thigh. Two motors, aligned in parallel to the hip joints, are used to apply hip extension and flexion torques. The power unit and the control hardware (sample rate of 1 kHz) were off-board during this study.

The shape of the assistance profile is inspired by the biological hip torque and is defined by four control parameters that determine the gait cycle timings of the transition from flexion to extension torque (*t*_1_), the extension torque peak (*t*_2_), the transition from extension to flexion torque (*t*_3_), and the flexion torque peak (*t*_4_). The timings are expressed in terms of the normalized stride time (ST), denoted as %_ST_ to avoid confusion with other quantities expressed as percentages. Phases in between the four control parameters are defined by half-cycle sinusoidal curves (Fig. 1(b)). Peak torques were fixed at 12 N m. In the zero-torque conditions, the torque setpoint was 0 N m throughout the gait cycle, resulting in minor resistance of the inactive exoskeleton due to the low backdrive torque of the quasi-direct drive actuators (absolute mean 1.1 N m^69^). In order to synchronize the assistance profile with the human gait, the vertical ground reaction force (GRF) from the instrumented treadmill was used to detect the timing of consecutive heel strikes. The gait phase was estimated in real-time by extrapolation based on the average stride time of the last five strides.

### Study design

This study includes data of 11 participants without gait impairments (four females and seven males, age 22.8 ± 2.6 years, body mass 71.3 ± 8.8 kg, height 1.79 ± 0.09 m, mean ± standard deviation). None of the participants had prior exoskeleton experience. All participants gave written informed consent before the experiment. The study received a positive vote from the Ethics Committee of Technische Universität Darmstadt (EK 38/2020) and was conducted in accordance with the principles outlined in the Declaration of Helsinki. The experimental protocol included a preparatory part and three main parts (Fig. 1(d)). Given that none of the participants had prior experience with exoskeletons, we conducted a familiarization period before the user-tuning procedure to establish their understanding of the exoskeleton system and its assistance profile. For this purpose, participants walked with the exoskeleton on the treadmill for about 5 min, starting with assistance set to zero and then gradually increasing the magnitude to the final value, while the timing control parameters were set to the initial values of the user-tuning procedure. Subsequently, they familiarized themselves with the functionality of the thumbstick remote control by making large adjustments to each of the parameters in turn while viewing the corresponding assistance profile on a monitor. In the user-tuning part, the participants tuned the four exoskeleton control parameters while walking on the instrumented treadmill, resulting in a user-tuned assistance profile. Afterward, we surveyed the SoA for two different walking conditions, namely inactive exoskeleton and user-tuned assistance. The last part was a metabolic evaluation of the user-tuned assistance profile. The treadmill speed was set to 1.25 m s^−1^ in all parts of the experiment, which is within the typical range of preferred walking speeds for adults^1^.

### User-tuning procedure

The participants tuned the assistance profile using a wireless thumbstick remote control while walking on the treadmill (Fig. 1(c)). A monitor in front of the participants displayed the previous assistance profile, the currently active profile, and the profile that was currently tuned. The currently selected control parameter was highlighted, and participants were able to adjust its timing by moving the thumbstick left or right. Moving the thumbstick up or down allowed for switching between the control parameters. The adjustments were only applied to the exoskeleton by clicking the thumbstick. There was no fixed order in which the parameters had to be tuned and it was possible to adjust multiple timings before applying the new profile, i.e., the participants freely explored the solution space. The user-tuning was terminated when the participants felt that they had found their preference, with no predefined time limit.

Prior to the user-tuning procedure, participants were instructed to tune the assistance to their preferred setting that reduces walking effort. Furthermore, they were instructed to try out the entire ranges of possible parameter timings and to test a new assistance profile for at least five to ten steps before trying a different setting. All participants started the user-tuning procedure with the same initial settings, i.e., *t*_1_ = 95%_ST_, *t*_2_ = 25%_ST_, *t*_3_ = 50%_ST_, *t*_4_ = 70%_ST_. The timing parameters could be adjusted with a resolution of 0.5%_ST_. In order to ensure technically feasible parameter settings, each distance between two adjacent control parameters had to be in the interval of 10%_ST_ to 40%_ST_. The extension and flexion peak timings were limited to the first and second half of the gait cycle, respectively. When a parameter limit was reached, the respective value turned red on the monitor.

### Metabolic evaluation

The effort of walking with the preferred assistance and the surrounding metabolic cost landscape were examined in four sessions. In each of these sessions, one of the four control parameters was varied around the preferred setting. Each session consisted of 3 min standing, 2 min warm-up, 4.5 min zero-torque condition serving as the metabolic baseline for the session, five 4.5 min assisted conditions (four variations and the user-tuned assistance) and 0.5 min standing, totaling up to a duration of 32.5 min per session. The timing variations were −8%_ST_, −4%_ST_, +4%_ST_, and +8%_ST_. Timing variations were inspired by studies that investigated the effect of hip assistance timing on metabolic cost^19,55,59^. Following each session, there was a brief break of a few minutes, and the participants had the option to extend this break if desired. The order of assisted conditions was randomized within each session to ensure variability and to prevent bias. Moreover, the sequence of the sessions was counterbalanced across all participants, minimizing any potential order effects. Participants were asked to refrain from food intake for two hours prior to the experiment.

### Sense of agency

Participants tested two conditions, namely zero-torque and the user-tuned assistance, without prior knowledge of what the exoskeleton would do. They were only instructed to “focus on how the interaction with the exoskeleton feels”. Each condition comprised 1 min of walking, followed by a SoA questionnaire (Table 1) during which the treadmill was halted. The order of the two conditions was counterbalanced across all participants.

**Table 1:** Sense of agency questionnaire

Item 1	The exoskeleton moved just like I wanted it to, as if it were obeying my will.
Item 2	I felt as if I was controlling the movements of the exoskeleton.
Item 3	I felt as if I was causing the movements of the exoskeleton.
Item 4	Whenever I moved my legs, I expected the exoskeleton to move in the same way.

Items adapted from Caspar et al.^41^.

### Data collection and analysis

All the measured data was collected in real-time on the computer that runs the exoskeleton control in order to assure the synchronization of the different measurements (sample rate of 1 kHz). GRFs were measured using an instrumented treadmill (ADAL 3D-WR, HEF Tecmachine, Andrézieux-Bouthéon, France). Heel strikes were detected from the vertical GRFs using a force threshold of 20 N. Stride time was calculated for each leg as the duration between two consecutive heel strikes. Walking effort was evaluated using indirect calorimetry (K5, COSMED, Rome, Italy). The metabolic cost of walking was estimated by fitting a first-order dynamic model to the breath-by-breath data measured in each condition. Net metabolic cost during walking was obtained by subtracting the standing metabolic cost, which was computed by averaging 3 min of standing data. The average metabolic costs in the zero-torque and the user-tuned conditions were obtained by calculating the mean over the respective conditions of all four sessions. Metabolic reduction was calculated relative to the zero-torque condition.

The user-tuning procedure was recorded as a time series of all settings tested. Based on this data, we calculated characteristics that describe the overall procedure as well as the discrete settings tested. The overall procedure is characterized by the total tuning duration, the number of settings tested, and the solution space explored during tuning (range of settings). The discrete settings are described by the differences in timings between two consecutive settings (exploration step size), and the time spent testing a setting before applying the next one (time per setting). Due to a technical issue, only the total tuning duration, the preferred control parameters, and the metabolic evaluation data were saved for participant P7. Thus, P7 is not included in the analysis of the user-tuning procedure.

The SoA questionnaire comprised four agency items adapted from Caspar et al.^41^, rephrased to align with the exoskeleton context (Table 1). For each statement, the participants expressed their agreement on a visual analog scale (VAS) ranging from “strongly agree” to “strongly disagree”. The VAS position was normalized to a numerical value from 0 to 1, and the values of the four items were averaged to obtain the SoA score.

Unless otherwise stated, averaging was done by calculating the mean, and uncertainties are indicated by the standard error. When analyzing the time per setting and the exploration step sizes within the tuning procedure, averages were calculated individually for each participant before averaging across participants in order to avoid bias due to the different numbers of settings tested. In this context, the average for each participant was calculated using the median in order to reduce the impact of extreme values. All calculations and analyses were performed using MATLAB R2023b (MathWorks, Natick, MA, USA) and jamovi 2.3.28^70^.

### Statistics

The net metabolic cost of zero-torque condition and user-tuned assistance were compared using a Wilcoxon signed-rank test, since the normality assumption of the paired differences had to be rejected as indicated by the Shapiro-Wilk test (*p* = 0.027). A potential influence of the timing variations on the metabolic cost reduction was investigated by performing a repeated measures ANOVA for each control parameter. The sphericity assumption was evaluated using Mauchly’s test. For *t*_1_, the assumption was violated, thus, the Greenhouse-Geisser correction was applied. Equivalence was tested using the two one-sided *t*-tests (TOST) procedure^65^. For each timing variation, we tested whether the 90% confidence interval of the mean paired difference was within the equivalence bounds of ±5% metabolic cost reduction. The equivalence bounds are based on observations in previous work^19,53^, where average reductions of about 5% were not statistically significant. The SoA scores of zero-torque and user-tuned assistance were compared using a paired samples *t*-test. Whiskers of box plots extend to the smallest and largest observations within one and a half times the IQR from the first and the third quartile, respectively.

## Data Availability

The datasets generated and/or analyzed in this study are available from the corresponding authors on reasonable request.
